# Comparison of Different Methods for Spatial Analysis of Cancer Data in Utah

**DOI:** 10.1289/ehp.10815

**Published:** 2008-04-25

**Authors:** Wayne Ball, Sam LeFevre, Lars Jarup, Linda Beale

**Affiliations:** 1 Environmental Epidemiology Program, Utah Department of Health, Salt Lake City, Utah, USA; 2 Small Area Health Statistics Unit, Imperial College London, London, UK

**Keywords:** cancer, exposure assessment, risk assessment, statistics

## Abstract

**Background:**

The standardized incidence ratio (SIR) and SaTScan software are used by the Environmental Epidemiology Program (EEP), Utah Department of Health, to investigate health concerns and exposures in Utah (USA). Recently, the EEP acquired the Rapid Inquiry Facility (RIF). The RIF enables access of additional dimensions of data, identifies potentially exposed populations, and computes disease rates and relative risk statistics for that potentially exposed population.

**Objective:**

In this article we present a comparison of the SIR, SaTScan, and RIF methodologies in an investigation of cancer rates in residents living over contaminated groundwater plumes near Hill Air Force Base (HAFB) in Utah.

**Methods:**

For this study, we used cancer data from the Utah Cancer Registry for cancers of the lung, kidney, and non-Hodgkin lymphoma. We used SIR and the RIF to investigate the cancer rate in a defined population within the study area during six consecutive 5-year time intervals (1975–2004). We used SaTScan and the RIF to explore the study area for clusters.

**Results:**

The RIF risk analysis and SIR are mathematically identical. SIR is set up and computed by programming SAS; the RIF risk analysis, on the other hand, is set up through four menu-driven steps. The RIF disease-mapping feature enhanced the interpretation of SaTScan results. We found kidney and lung cancer to be statistically elevated for the potentially exposed population for one and two periods, respectively. SaTScan found two clusters, one outside the potentially exposed population and one that included a portion of that population.

**Conclusion:**

The RIF is an easy-to-use and useful tool that extends the ability of the investigator to conduct analysis of disease rates and interpret the findings.

The Environmental Epidemiology Program (EEP), Utah Department of Health (UDOH), investigates environmentally related health concerns. The EEP has used the standardized incidence ratio (SIR) to investigate small-area population health risks, incorporating the time factor into these analyses through the use of consecutive periods. SIR and this approach to spatial analysis are known to be problematic (da Silva et al. 2006; Richardson et al. 2004). The scan statistic implemented in the SaTScan software (Kulldorff 2006) is an increasingly popular adjunct for investigating geospatially oriented health concerns (SaTScan 2006). However, proper use of the SaTScan statistic requires some data manipulation outside the capabilities of software based on geographic information systems (GIS), such as Economic and Social Research Institute ArcGIS (Redlands, CA, USA). Recently, the EEP acquired the Rapid Inquiry Facility (RIF) application from the Small Area Health Statistics Unit, Imperial College London, to improve capacity and efficiency to conduct public health investigations of environmental related diseases such as cancer.

The RIF is a functional extension of the ArcGIS version 9 GIS software. The RIF enables access of additional dimensions of data, identifies potentially exposed populations by proximity to geographically defined environmental hazards, and computes the disease rate and relative risk (RR) statistics for that potentially exposed population (Jarup 2004). The advantages of the RIF over traditional methods are the integration of a comprehensive database linking health, population, environmental, and covariate data and the use of Bayesian methodologies in the calculates of the disease rate and RR statistics (Jarup 2004).

Hill Air Force Base (HAFB) is an active Air Force base and logistics support, maintenance, and storage depot located on approximately 6,670 acres in Davis and Weber Counties in Utah. The base, situated on a plateau roughly 300 feet above the valley floor, sits over two shallow aquifers. Depot operations from 1950 until the present have resulted in contamination of groundwater under the base with trichloroethylene (TCE) and related products and plumes of contaminated groundwater migrating into the residential areas surrounding the base. Investigation of the groundwater contamination began in 1976 and resulted in the placement of HAFB on the National Priority List in July 1987. Controls and cleanup activities were started in 1998 [U.S. Agency for Toxic Substances and Disease Registry (ATSDR) 2003]. The EEP previously completed studies of cancer rates in residents living over or near the groundwater plumes (LeFevre and Ball 2005; Williams et al. 2003). We used the HAFB study as a test environment to compare the SIR method with the SaTScan and RIF methods.

Biologically relevant cancers associated with exposure to the groundwater contaminates are those of the central nervous system, esophagus, kidney and renal pelvis, liver and intrahepatic bile duct, and lung and bronchus, as well as multiple myeloma and non-Hodgkin lymphoma (ATSDR 1994, 1997a, 1997b, 2003). For brevity, we present here the investigation of cancers of the kidney and renal pelvis, lung and bronchus, and non-Hodgkin lymphoma.

## Materials and Methods

**Figure 1:**
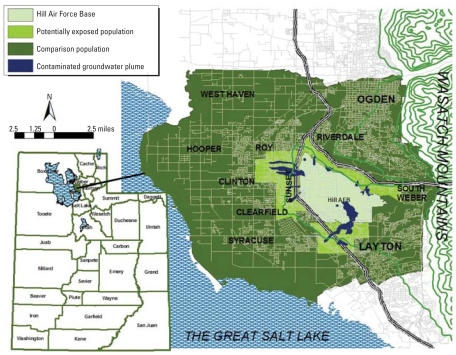
Map of Hill Air Force Base (HAFB), Utah, and surrounding communities, presenting contaminated groundwater plumes and the potentially exposed population in the study area.

The study area consisted of 11 ZIP code areas in northern Davis and southern Weber Counties that included communities surrounding and contiguous with HAFB (Figure 1). We used commercially available U.S. 2000 census data to obtain the population, median income level, and percentage of the population with a residential tenure (RT) > 5 years for the 143 census block groups (CBGs) contained in the study area (Geolytics 2008a, 2008b). The CBG is the smallest census geography at which all census factors are tabulated. The CBG includes 600–3,000 people, with an optimum of 1,500 persons (U.S. Census Bureau 2005). The study population contained approximately 247,500 persons. HAFB provided UDOH with concentration boundary data for 12 groundwater plumes contaminated with TCE and related compounds (U.S. Air Force 2001). Limited information was available regarding the history, meander, true extent, and potential routes of exposure (LeFevre and Ball 2005). Proximity-based exposure assessment is an increasingly popular approach (for discussion, see Maantay 2002). The assignment criteria for the potentially exposed population included the total population of CBGs for which any portion of the CBG was within 400 m of any one of the contaminated groundwater plumes at the 5–10 μg/L concentration boundary (LeFevre and Ball 2005). Approximately 53,500 persons resided in the 32 CBGs with potentially exposed populations. The remainder of the study area was the comparison population. We obtained cancer data on first primary cases of cancer from 1973 through 2004 from the Utah Cancer Registry and geocoded nearly all (97.9%) of the cases to the appropriate year 2000 CBGs in the study area (Utah Cancer Registry 2006). For comparison of the methods, we used data from 1975 through 2004 organized into six consecutive 5-year analytical periods (1975–1979–1980–1984–1985–1989–1990–1994–1995–1999, and 2000–2004) and the total 30-year study period (1975–2004). We conducted analysis for age and sex standardization and with one or both of two additional covariates, socioeconomic status (SES) and RT. We combined age and sex into an age–sex code and included this with SES and RT as covariates for analysis in all methods. We defined SES by the ranking CBG median income (6 ranks), and RT as the ranking of the percentage of CBG population > 5 years of age who had lived at the same address for > 5 years (10 ranks). The demographics of the potentially exposed and comparison population are similar with respect to age, sex, and race/ethnicity distribution. Both the SES and RT had a spatial structure to the distribution of the rank values as determined by Moran’s *I* statistic. The median income represented by SES (*I* = 0.10, *p* < 0.0001) for the study area was generally higher on the east side of the study area and lowest to the north. The mobility, represented by RT (*I* = 0.03, *p* < 0.001), was highest near the base and lower with increasing distance from the base.

We calculated SIR for the potentially exposed population for each 5-year analytical period for three cancer sites used for method comparison purposes: lung and bronchus (1,167 cases in the study area between 1975 and 2004), non-Hodgkin lymphoma (566 cases), and kidney and renal pelvis (267 cases). The ratio was standardized on *a*) age and sex; *b*) age, sex, and SES; *c*) age, sex, and RT; and *d*) age, sex, SES, and RT. The EEP uses SAS (version 9.1; SAS Institute, Inc., Cary, NC, USA) to assign case and census population data to the correct study group, aggregate the data, and compute the metrics.

The RIF application (version 3.0; Rapid Inquiry Facility, London, UK) operating on ArcGIS (version 9.2) provides two features, risk analysis and disease mapping. The risk analysis allows the comparison of the aggregated CBGs comprising the potentially exposed population with the aggregated CBGs comprising the comparison population for a user-defined study period. RIF computes a direct standardized rate based on *a*) age by sex and *b*) age by sex with other covariates, and an indirect standardized RR based on *a*) age by sex and *b*) age by sex with other covariates (Small Area Health Statistics Unit 2006). We used RIF risk analysis to compute RR for the potentially exposed population for each of the three cancer types, for each analytical period, and for each combination of covariates.

The RIF disease-mapping feature compares the cancer rate for each CBG in the study area with a comparison rate derived from a user-defined comparison population for a specified study period. For this study, we used the total study area rate as the comparison rate and six consecutive 5-year analytical periods along with the 30-year study period for the time dimension. The disease-mapping feature computes both smoothed and nonsmoothed standardized rates and RRs. We used the same covariates for standardization as described above. We identified clusters by visual inspection of maps.

We used SaTScan statistical software (version 7.0.1) as a third method (Kulldorff 2006). We used the SaTScan space–time analysis, using the Poisson probability model and constrained to clusters no larger than 50% of the population at risk and 50% of the study period (1975–2004), to locate potential circular and elliptical cluster areas (Kulldorff 1997). We included no other spatial or temporal adjustments. We computed significance using 999 Monte Carlo simulations.

For brevity, we describe only the analysis of the lung and bronchus cancers in the RIF disease mapping and SaTScan comparison. We considered clusters found through the RIF disease mapping or the SaTScan applications relevant to the exposure of concern (the TCE plumes) if 51% of the cluster area was within the potentially exposed population.

## Results

In this investigation we compared RIF risk analysis with the SIR method used by EEP to evaluate cancer risk in a defined population, and RIF disease mapping with SaTScan to explore for potential clusters of interest. The RIF risk analysis RR is mathematically the same as SIR. The EEP uses desktop SAS to organize and query cancer and population data, assign exposed and comparison populations to a study, and compute SIR. This process requires an understanding of SAS programming. An immediate advantage of the RIF application is the four-step menu-driven process to accomplish these tasks, which does not require an understanding of SAS.

Table 1 presents a comparison of the risk analysis RR for each of the three cancer types for each analytical period and for each combination of covariates. The incidences of lung and bronchus cancer and kidney and renal pelvis among the potentially exposed population were both statistically elevated for one analytical period (1995–1999). Those cancer rates remained a concern when accounting for the additional covariates (SES and RT). An additional analytical period (1980–1984) became statistically significant for elevated lung and bronchus cancers when we included the additional covariates.

**Figure 2:**
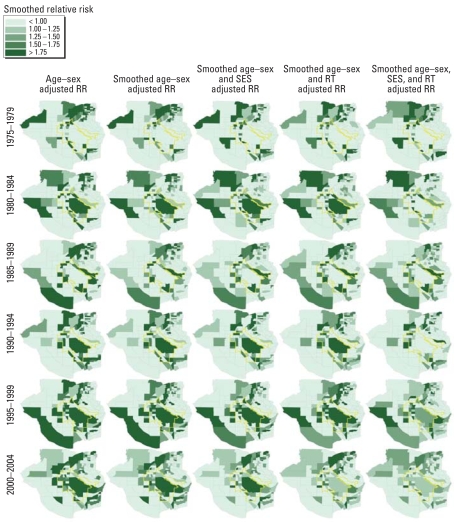
Graphic representation of nonsmoothed and smoothed CBG RRs for each 5-year analytical period and for combinations of covariates from 1975 through 2004 for cancers of the lung and bronchus.

An advantage of the RIF is the ability to quickly explore disease status in a number of dimensions. In this study, we explored five dimensions: geography, time, demography and other population covariates, cancer site, and smoothing. Figure 2 attempts to present RIF disease-mapping results in four (geography, time, covariates, and smoothing) of those dimensions (some only partially presented) for lung and bronchial cancers. For this graphic, the scaling of the RR demonstrates the effects of smoothing and covariate inclusion on the RR between 1.0 and 2.0.

**Figure 3:**
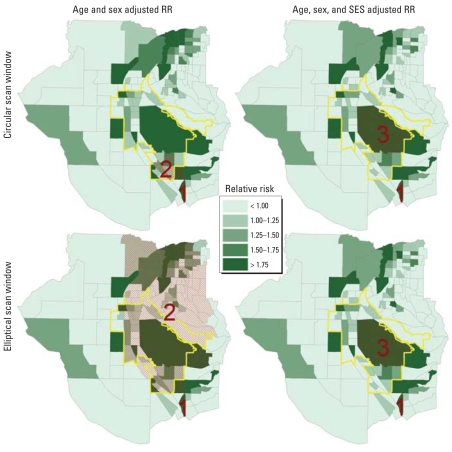
Potential clusters of CBGs with statistically significant elevated rates of lung and bronchus cancer identified by SaTScan (red cross-hatching) overlaying the graphical representation of the non-smoothed 30-year study-period RR computed by the RIF disease mapping (green shading). Yellow outlines indicate location of the potentially exposed population. Numbers indicate clusters identified in Table 2.

Table 2 and Figure 3 present results for SaTScan. Figure 3 also presents disease mapping by the RIF. SaTScan identified two statistically significant clusters in the study area. Cluster 1 was consistently located in the same CBG for the same time period (1994–2003) and includes off-base housing for HAFB. Cluster 2 was associated with the potentially exposed population. The utility of using the RIF disease mapping in concert with SaTScan is demonstrated by the graphic of the second cluster using an elliptical window. Here, SaTScan appears to have extended the circular cluster by aggregated several areas of apparent clustering (demonstrated by visual inspection of the RIF results) occurring during the same general time period (1981/1983–1987). When we included SES in the analysis, this second cluster was no longer significant. Instead, a third cluster (distinct in time from cluster 2) located on HAFB itself is present. When we included RT in the analysis, either by itself or in combination with SES, no statistically significant clusters were located by SaTScan.

## Discussion

In this study, we compared three methods to investigate the incidence of cancer among residents living over contaminated shallow groundwater plumes originating from HAFB in Davis and Weber Counties, Utah, between 1975 and 2004. Cancers typically have long latency periods between the probable causal events and disease manifestation. Further, the causality of cancer is complex, and the time of diagnosis may be subject to ability to seek medical screening as well as onset of clinical manifestation. Potential exposure assessment can be confounded by behavioral risks, genetic propensity, and unknown environmental risks, as well as the dynamics (intensity and duration) of the studied environmental exposure. Applying methods that allow the exploration of the spatial and temporal structure of disease allows the investigator to further identify potential populations and factors of interest for further investigation. In this investigation, we discovered an excess risk of lung and bronchial cancer and of kidney and renal pelvis cancer associated with the potentially exposed population. However, the analysis does not necessarily link the risk to the exposure. Including covariates that measure alternative explanations improves linkage. The population living on HAFB and in the communities surrounding HAFB is dominated by active-duty military personnel and Department of Defense employees or contractors who have comparatively short RTs and differing lifestyle behaviors than the population residing farther from the base and within the study area (LeFevre and Ball 2005). This study included two covariates, as indirect measures of those population characteristics, in an attempt to control for those features. We used SES as a surrogate for lifestyle, including the use of tobacco. We used RT, which is a measure of population mobility, as a surrogate for potential exposure duration. We applied both covariates only on the ecologic scale. A weakness of the study is how well the covariates represent the population feature of concern. For example, the SES assumes an inverse correlation between income and tobacco use behavior. The relationship may be comparatively true for enlisted military personnel but is unlikely to be true for defense employees and contractors. Implementing more controls in the calculation of the risk measures can sometimes lead to increased and more profound significant findings (Elliott and Wartenberg 2004).

The EEP has used SIR as the method to investigate cancer incidence. SIR is easy to compute, is straightforward to interpret, and has a history of use in public health investigations. SIR depends on the ability of the investigator to define an at-risk population and is problematic with respect to distribution assumptions (da Silva et al. 2006; Richardson et al. 2004). SIR is mathematically identical to the RIF risk analysis, with the exception of the choice of methods for computing confidence intervals. The EEP uses SAS software to organize case and population data for an investigation and to compute SIR, requiring the investigators to have experience in SAS programming. The RIF, on the other hand, is a menu-driven tool that allows investigators to set up an investigation in four steps.

The SaTScan method is an increasingly popular adjunct for exploring the spatial and temporal distribution of disease (SaTScan 2006). The EEP has used SaTScan to confirm investigations of disease and to further explore disease patterns. This method compares all possible aggregations of neighboring populations and time slices with the rest of the study area and orders those aggregations on the likelihood that a cluster of cancer incidence exists within the aggregated area and time. The method has been implemented in an easy and intuitive computer application. An advantage of the SaTScan method is its ability to uncover the spatial and temporal location of clusters and to use a variety of distribution models, depending on the available data (Kulldorff 2006; SaTScan 2006). However, the method may uncover clusters that are not relevant to the exposure. Currently, SaTScan does not operate within popular GIS applications such as ArcGIS. Data have to be exported for SaTScan, and results have to be linked back to the GIS data for visualization.

The RIF provides two mapping features operating within the ArcGIS environment. The risk analysis feature can be used to evaluate risk for a defined population of an aggregated small area (e.g., CBG), similar to SIR. The RR produced by the RIF is intuitive to investigators for interpretation. The disease-mapping feature supports exploratory investigations and overcomes distribution problems by employing Bayesian methodology (Jarup 2004). The present investigation presents an example of the utility of using RIF disease mapping with SaTScan.

The findings of this investigation demonstrate the utility of the RIF as a tool for both investigating the risk of disease in a defined population and exploring the distribution of disease in conjunction with other exploratory tools such as SaTScan.

## Figures and Tables

**Table 1:** Five-year RR for selected cancer incidences among residents living near contaminated shallow groundwater plumes in communities surrounding HAFB, 1975–2004.

		RR (95% confidence interval)			
Cancer/period	Observed cases	Age and sex	Age, sex, and SES	Age, sex, and RT	Age, sex, SES, and RT
Cancers of the lung and bronchus					
1975–1979	17	0.72 (0.42–1.15)	1.27 (0.72–1.98)	0.97 (0.56–1.55)	1.41 (0.82–2.25)
1980–1984	40	1.26 (0.90–1.71)	1.46 (1.04–2.01)	1.45 (1.03–1.97)	1.51 (1.07–2.07)
1985–1989	41	1.32 (0.95–1.79)	1.34 (0.96–1.82)	1.30 (0.93–1.76)	1.37 (0.98–1.86)
1990–1994	38	0.97 (0.68–1.33)	0.90 (0.63–1.25)	0.85 (0.60–1.16)	1.02 (0.71–1.42)
1995–1999	59	1.52 (1.16–1.97)	1.37 (1.04–1.78)	1.37 (1.04–1.77)	1.48 (1.12–1.92)
2000–2004	57	1.24 (0.94–1.61)	0.98 (0.74–1.28)	1.03 (0.78–1.33)	1.09 (0.82–1.43)
Cancers of the kidney and renal pelvis					
1975–1979	6	0.92 (0.34–2.01)	0.88 (0.28–2.05)	1.04 (0.38–2.26)	0.70 (0.23–1.63)
1980–1984	3	0.62 (0.13–1.81)	0.51 (0.06–1.83)	0.72 (0.15–2.09)	0.50 (0.06–1.82)
1985–1989	7	1.01 (0.41–2.09)	0.86 (0.35–1.78)	0.98 (0.39–2.01)	0.89 (0.36–1.84)
1990–1994	14	2.23 (1.22–3.75)	2.95 (1.57–5.04)	2.20 (1.20–3.69)	3.17 (1.69–5.42)
1995–1999	13	1.09 (0.58–1.86)	1.01 (0.57–1.91)	1.10 (0.58–1.88)	1.22 (0.63–2.13)
2000–2004	14	0.93 (0.51–1.55)	0.86 (0.47–1.45)	0.79 (0.43–1.32)	0.68 (0.37–1.14)
Non-Hodgkin lymphoma					
1975–1979	7	0.76 (0.31–1.57)	0.91 (0.36–1.87)	1.14 (0.46–2.34)	1.28 (0.52–2.65)
1980–1984	11	0.74 (0.37–1.32)	0.90 (0.45–1.61)	0.84 (0.42–1.50)	0.94 (0.47–1.67)
1985–1989	20	1.25 (0.76–1.93)	1.21 (0.74–1.86)	1.55 (0.95–2.40)	1.44 (0.88–2.22)
1990–1994	21	1.15 (0.71–1.76)	1.20 (0.74–1.86)	1.28 (0.79–1.96)	1.50 (0.92–2.32)
1995–1999	28	1.44 (0.96–2.08)	1.32 (0.86–1.94)	1.21 (0.81–1.75)	1.16 (0.76–1.71)
2000–2004	29	0.99 (0.66–1.42)	0.87 (0.58–1.26)	0.95 (0.64–1.36)	0.91 (0.60–1.31)

**Table 2:** Significant clusters found by the SaTScan software (version 7.0.1) for lung and bronchial cancers in the HAFB study area, 1975–2004.

Window shape	Covariates included in analysis	Cluster label	Time period	Population	No. of cases	Expected no. of cases	RR	*p*-Value
Circular	Age, sex	1	1994–2003	623	11	1.51	7.46	0.014
Circular	Age, sex	2	1983–1987	8,794	17	4.16	4.21	0.050
Elliptical	Age, sex	1	1994–2003	623	11	1.51	7.45	0.025
Elliptical	Age, sex	2	1981–1987	44,320	74	40.17	2.01	0.043
Circular	Age, sex, SES	1	1994–2003	623	11	1.36	8.26	0.011
Circular	Age, sex, SES	3	1990–2003	5,131	9	0.92	9.93	0.024
Elliptical	Age, sex, SES	1	1994–2003	623	11	1.36	8.26	0.017
Elliptical	Age, sex, SES	3	1990–2003	5,131	9	0.92	9.93	0.037
